# Wellbeing for all: Indigenizing theories and measures of wellbeing for equitable sustainability

**DOI:** 10.3389/fpsyg.2022.979109

**Published:** 2023-01-26

**Authors:** Joanne Qina‘au, Mapuana C. K. Antonio

**Affiliations:** ^1^Department of Psychology, College of Social Sciences, University of Hawai'i at Mānoa, Honolulu, HI, United States; ^2^Native Hawaiian and Indigenous Health, Office of Public Health Studies, Thompson School of Social Work and Public Health, University of Hawai'i at Mānoa, Honolulu, HI, United States

**Keywords:** wellbeing, Indigenous, theory, measurement, public policy, sustainability, equity

## Introduction

In the midst of war on multiple fronts, a global pandemic, social strata rife with inequities, the slow looming impacts of climate change, and a multi-level sustainability crisis, ecological and psychosocial resilience are vital to individual and collective wellbeing. At the international level, wellbeing continues to be a major priority, as evidenced by the United Nations' (UN) Sustainable Development Goals (SDGs), particularly Goal 3, ensuring healthy lives and wellbeing for all. Given the clear connection between large-scale ecological events and personal wellbeing, how might we best approach wellbeing for all? In alignment with Indigenous ontologies, wellbeing is *ecological* and encompasses physical, mental, interpersonal, familial, community, spiritual, and environmental levels—emphasizing *relationality, interconnectedness*, and the *cyclical nature* of wellbeing processes. These perspectives of wellbeing may provide balanced solutions to the UN SDGs and align with the One Health policy and intervention approach, a transdisciplinary pluralistic perspective aimed at serving people, animals, and their environments (Hillier et al., 2021). As such, we offer three opinions: (1) Indigenized views of wellbeing should be leveraged as a sustainable and powerful option in realizing wellbeing for all; (2) In service of wellbeing for all, communities might also consider measuring wellbeing for public policy and design measures based on their unique context; (3) Island nations and communities are most acutely vulnerable to climate change, and their wellbeing must be made a priority to meet UN SDGs.

## Climate change is at our doorstep

Global climate change is projected to have acute and chronic deleterious effects on psychological wellbeing for individuals across the world (Swim et al., 2009; Doherty and Clayton, 2011). Anxiety reactions to climate change are on the rise (Clayton, 2020), as are negative impacts on the psychological wellbeing of children (Burke et al., 2018). Given the significant and undeniable role of environmental health on community and individual wellbeing, tools designed to reliably assess the complex levels and dimensions of wellbeing should consider the framework of ecological wellbeing (Lambert et al., 2020; Betley et al., 2021). Such a framework is ideal in meeting UN SDGs by considering effects of environmental health on humans, emotional and spiritual relationships to the environment, allowing for a more equitable form of wellbeing for all.

## Opinion 1: Wellbeing for all can be achieved through indigenized wellbeing

An ecological view of wellbeing was proposed by Hippocrates in 500 BC (*On Airs, Waters, and Places*) in which he posited natural surroundings, housing conditions, and social patterns of a community greatly influences health and wellbeing (Barton, 2016). Hippocrates' ecological framing is on par with many traditional Indigenous worldviews that honor symbiotic relationships between humans and the natural world (Murietta, 2015). An example of how this may be reflected in policy can be found in the Whanganui River legislation, giving it legal personhood (Charpleix, 2018). Although variation exists across and within Indigenous communities in their views on wellbeing, their similarities shed light on achieving wellbeing for all.

Indigenous perspectives often emphasize the ecological framing that are innately relational—our planet is seen as a spiritual mother figure, whom humans have a responsibility to protect as stewards and must live in harmony (McAvoy et al., 2003). In the same way healthy humans form important attachment bonds with their human mothers, so too do Indigenous communities with their spiritual mothers, Mother Earth. Imagine for a moment, how vastly different our ecological wellbeing might be if we related to land as mother, sky as father, and plants as siblings, as in the Hawaiian tradition (Trask, 2004; Kikiloi and Graves, 2010). This ontology goes beyond proximity approaches to framing climate change (Brügger et al., 2015) and elevates it to the relational level (e.g., McCubbin et al., 2012).

Since its first Conference on Human Environment in 1972, the UN international community acknowledges the urgent need to maintain balance and harmony between nature and mankind (Kuriansky et al., 2015). In 2012, the U.N. established rights for Mother Earth (Papahānaumoku in Hawaiian cosmologies), emphasizing the importance of living in balance with nature (United Nations, 2012). These rights were re-endorsed in 2014, when the UN recognized publicly building “governance structures in which nature is treated in partnership with humankind” (United Nations, 2014) as imperative (for a review of measures of nature connectedness see Keaulana et al., 2021). Harmony and balance across levels of wellbeing are of increasing interest to the wellbeing research community, as evidenced in the World Happiness Report (Helliwell et al., 2022). A wellbeing framework that is built on the foundations of balance or being *pono* (righteous; see Kaholokula, 2017), which inherently encourages equity—equitable relationships with nature, equitable distribution of resources, and equitable access to wellbeing.

Indigenized wellbeing recognizes the interconnectedness and the cyclical nature of all things, a view that would no doubt advance the platform of wellbeing for all. Interconnection is emphasized across (e.g., balanced individual wellbeing is connected to balanced community wellbeing) and within levels (e.g., psychological challenges in the family unit are connected to family functioning). Cycles are emphasized across dimensions and time, creating an imperative for adults to engage in wellbeing-supportive behaviors in ways that honor ancestors and provide for future generations (Meyer, 2001; McGregor et al., 2003).

For Indigenous communities, these aspects of ontologies and epistemologies are interwoven into spirituality and praxis—indeed, culture, spirituality, and place attachment are inextricably intertwined with identity, and therefore, wellbeing (Durkalec et al., 2015; Oppong et al., 2020). However, one need not be of Indigenous spiritual orientations to leverage the power of belief systems that bring about wellbeing for all. Spirituality is the capacity for self-transcendence—realization that is greater than the self (e.g., sacred) and the search for “connectedness, meaning, purpose, and contribution” (Fleming and Ledogar, 2008). The UN international community named spirituality as core to its initial mission. The second Secretary-General of the UN, H.E. Dag Hammarskjold explicitly stated, “We can only succeed in achieving world peace if there is a Spiritual Renaissance on this planet” (The Spiritual History of the United Nations, 2017). Spiritual views emphasize that what we do and how do it matter, not only to us, but to our future generations, our loved ones, plants and animals, and even folx we do not know but are connected to through the air we breathe, the Earth we live on, and our basic human experience. Indigenized wellbeing provides a non-anthropocentric blueprint for equity, balance, stewardship, and relationship to the environment that makes wellbeing for all possible and sustainable.

## Opinion 2: Wellbeing for all begins with communities

Wellbeing measures designed from Indigenous worldviews and validated with multiple groups are available (e.g., McCubbin et al., 2013) and several research teams are working on developing additional measures (e.g., Dudgeon et al., 2017). However, to ensure the unique influences of local culture and context underlying conceptualizations of wellbeing, a participatory approach may identify more reliable indicators of wellbeing assessments (King et al., 2014). Participatory methods often result in accurate information about resources, vulnerability, and resilience as well as findings that reflect the values and priorities of community, enhancing the success of supporting policy measures (Glaser, 2003). Through this approach, a space is created for community members to reflect on concepts of wellbeing, add their voices to the process, co-learn alongside researchers, empower and further motivate them toward realization of wellbeing in their personal lives, and make possible the development of greater equity and autonomy (Diener, 2009; Kia-Keating and Juang, 2022). Although participatory methods have potential drawbacks (e.g., cost, precarious validity, potential to ignore less vocal community members, and anticipation of assistance by the community from the researchers), they have been used to successfully define wellbeing indicators within the context of ecosystems for communities across the globe (see King et al., 2014, for a review).

Assessing wellbeing for public policy comes with a plethora of advantages for legislators and administrators of states: enhancing economic analyses (e.g., assessing the cost-benefit dynamic of externalities such as the way influences on wellbeing may dictate income and spending); evaluating and improving public goods and services; identifying characteristics of desirable living and working spaces; and advancing the definition of a good society (Adler and Fleurbaey, 2016). While it has been suggested a psychological Heisenberg principle may come into play (i.e., the object being measured changes due to the act of measurement), Diener (2009) posit that the very act of measuring wellbeing on larger scales will consequently raise awareness of and motivation to enhance wellbeing in individuals and communities.

The following countries and cities have already implemented procedures to use wellbeing assessments—or some near equivalent, like happiness, or life satisfaction—for public policy: United Kingdom, New Zealand, Germany, Canada, Jalisco, and Santa Monica. Similarly, the United Arab Emirates, Venezuela, and Ecuador have appointed ministers of happiness. Notably, Bhutan's Gross National Happiness Project offers a model of how a collaboratively formulated battery of wellbeing measures can safeguard locally-defined cultural and spiritual priorities while providing policy makers with important data regarding wellbeing within the framework of global climate change.

## Opinion 3: Ecological and equitable wellbeing for island nations and communities

The present opinion piece is written from the positionality of Kānaka Maoli scholars who were born and raised in Hawai‘i, and whose current professional work focuses on the wellbeing of their island communities. As such, we make this offering with the mindset that climate change is a global issue that differentially impacts island nations and communities, and thus, draw our attention to solutions for island communities to elicit positive change.

While it is well-established that “environmental deterioration significantly influences the social wellbeing and cultural stability of traditional inhabitants” (Ozkan and Schott, 2013, p. 1), Indigenous communities from island nations are particularly vulnerable (Mimura et al., 2007; Swim et al., 2009; McIver et al., 2015; World Health Organization, 2015). Yet, Pacific Island nations and communities do not appear in United Nations (2012) *World Happiness Report*, a review of happiness levels for 146 nations. It is critical that island nations have a place at the global wellbeing measurement table.

Current measures of wellbeing may not be sufficient for meeting SDGs in many localities, and efforts are currently underway in island nations to contextualize wellbeing measurement (see Sterling et al., 2020). Politicians and legislators in island nations at city, state, and national levels might consider how best to integrate local cultural values, SDGs, and an Indigenized approach to wellbeing to move toward wellbeing for all. Leaders of cultural groups that find themselves in complex political situations, such as long-term illegal occupation, might consider designing wellbeing measurements for their unique communities, such as the Native Hawaiian lāhui (e.g., Kukulu Kumuhana Planning Committee, 2017). By the same token, stakeholders who play an influential role in political decisions should consider Indigenized and ecological measures of wellbeing, especially for health and environmental assessments.

## Final thoughts

With the global climate clock ticking away, it is imperative to explore and understand wellbeing for all. We posit that leveraging the power of an Indigenous worldview in conceptualizing and measuring wellbeing provides a clear blueprint for pursuing this worthy goal. Indigenizing wellbeing brings the environmental level to the forefront, reminding us of our undeniable relationship to nature and emphasizing our interconnectedness across levels. Indigenized wellbeing emphasizes a cyclical approach to individual responsibility to future generations—through this process, we recognize that *we ourselves are future ancestors*. Underscoring the vital need for balance, not only in this stewardship, but in our distribution of material resources, Indigenizing wellbeing prioritizes issues of equity.

An Indigenized approach to wellbeing is best coupled with locally informed indicators, gathered through participatory methods, which speak to a community's unique context and culture. Integrating psychometrically sound wellbeing measurement with policy and future interventions may also play a vital role in achieving the UN SDGs, including goals that aim to ensure healthy lives and wellbeing for all. Whatever path future researchers, policy makers, and change-makers may choose, the time is now for contextually relevant wellbeing assessment with island nations and communities.

## Author contributions

Both authors listed have made a substantial, direct, and intellectual contribution to the work and approved it for publication.
